# Clinical and neuroimaging review of monogenic cerebral small vessel disease from the prenatal to adolescent developmental stage

**DOI:** 10.1007/s11604-023-01493-0

**Published:** 2023-10-17

**Authors:** Mikako Enokizono, Ryo Kurokawa, Akira Yagishita, Yasuhiro Nakata, Sho Koyasu, Hiroshi Nihira, Shigeko Kuwashima, Noriko Aida, Tatsuo Kono, Harushi Mori

**Affiliations:** 1 Department of Radiology, Tokyo Metropolitan Children’s Medical Center, 2-8-29 Musashidai, Fuchu, Tokyo, 183-8561 Japan; 2 Department of Radiology, Graduate School of Medicine, The University of Tokyo, Bunkyo-ku, Tokyo Japan; 3 Department of Neuroradiology, Tokyo Metropolitan Neurological Hospital, Fuchu, Tokyo Japan; 4 Department of Diagnostic Imaging and Nuclear Medicine, Kyoto University, Sakyo-ku, Kyoto Japan; 5 Department of Pediatrics, Kyoto University, Sakyo-ku, Kyoto Japan; 6 Department of Radiology, Dokkyo Medical University, Shimotsuga-gun, Tochigi Japan; 7 Department of Radiology, Kanagawa Children’s Medical Center, Yokohama, Kanagawa Japan; 8 Department of Radiology, School of Medicine, Jichi Medical University, Shimotsuke, Tochigi Japan

**Keywords:** Cerebral small vessel disease, Monogenic, Hereditary, Pediatric, Cerebral calcification

## Abstract

Cerebral small vessel disease (cSVD) refers to a group of pathological processes with various etiologies affecting the small vessels of the brain. Most cases are sporadic, with age-related and hypertension-related sSVD and cerebral amyloid angiopathy being the most prevalent forms. Monogenic cSVD accounts for up to 5% of causes of stroke. Several causative genes have been identified. Sporadic cSVD has been widely studied whereas monogenic cSVD is still poorly characterized and understood. The majority of cases of both the sporadic and monogenic types, including cerebral autosomal-dominant arteriopathy with subcortical infarcts and leukoencephalopathy (CADASIL), typically have their onset in adulthood. Types of cSVD with infantile and childhood onset are rare, and their diagnosis is often challenging. The present review discusses the clinical and neuroimaging findings of monogenic cSVD from the prenatal to adolescent period of development. Early diagnosis is crucial to enabling timely interventions and family counseling.

## Introduction

Cerebral small vessels consist of two components: first, the vasoganglion deriving from the leptomeninges covering the subarachnoid space and the convex surface of the brain; and second, perforating arteries originating in the anterior, middle, and posterior cerebral arteries, which supply the subcortical parenchyma. Cerebral small vessels are essential for maintaining adequate blood flow to the subsurface brain structures [1].

Cerebral small vessel disease (cSVD) refers to a group of pathological processes with various etiologies affecting the small vessels of the brain, including small arteries and arterioles but also capillaries and venules [2], which are commonly 50–400 mm in length [1]. To understand better the pathogenesis of cSVD, it is necessary to consider how capillary endothelial cells and pericytes interact with astrocytes, oligodendrocytes, and microglia, which comprise the neuro­glio-vascular unit, where cells interact to regulate the entry of fluid and nutrients into the interstitium, manage blood supply, maintain and repair myelin, clear fluid and waste, and maintain the interstitial milieu for appropriate cell function [3]. Neuro-glio-vascular unit dysfunction plays a key role in the initiation and progression of cSVD.

Several manifestations of cSVD have been identified in humans, including blood–brain barrier (BBB) dysfunction, impaired vasodilation, vessel stiffening, dysfunctional blood flow, interstitial fluid drainage, white matter rarefaction, ischemia, inflammation, myelin damage, and secondary neurodegeneration in global brain effects [4–7]. Many affected individuals exhibit no symptoms in the early stages of this condition, but brain damage can result in sudden-onset stroke, cognitive decline, dementia, gait and balance problems, sphincter dysfunction, and psychiatric disorders [8, 9].

The neuroimaging features of cSVD include small, subcortical infarcts, lacunar infarcts, white matter hyperintensities, enlargement of perivascular spaces, microbleeds, superficial siderosis, large hemorrhages, calcification, and brain atrophy [2, 3, 10]. Because small vessels cannot currently be visualized in vivo, the term, small vessel disease is frequently used to describe parenchymal lesions rather than the underlying small vessel alterations. Sensitive MRI techniques can reveal pathological alterations occurring in the so-called normal-appearing white and gray matter [4, 5].

Pantoni has classified cSVD into the following categories according to pathological cerebrovascular changes [2]: Type 1, arteriolosclerosis (or age-related and vascular risk factor-related small vessel diseases); Type 2, sporadic and hereditary cerebral amyloid angiopathy (CAA); Type 3, inherited or genetic small vessel diseases distinct from cerebral amyloid angiopathy; Type 4, inflammatory and immunologically mediated small vessel diseases; Type 5, venous collagenosis; and Type 6, other small vessel diseases, such as post-radiation angiopathy. These various, pathological changes result in damage to the brain parenchyma, including neuronal apoptosis, diffuse axonal injury, demyelination, and loss of oligodendrocytes.

Most cases of cSVD are sporadic, with the age-related and hypertension-related types and CAA being the most prevalent forms. Monogenic cSVD accounts for up to 5% of all causes of stroke [11]. Several causative genes have been identified [12–15]. Monogenic cSVD, the most common type, is an autosomal-dominant, cerebral arteriopathy characterized by subcortical infarcts and leukoencephalopathy (CADASIL) arising from *NOTCH3* gene mutations [16]. Sporadic cSVD has been widely studied whereas monogenic cSVD is still poorly characterized and understood. The majority of cases of both the sporadic and monogenic types, including CADASIL, typically have their onset in adulthood. Forms of the disease manifesting in infancy and childhood are rare, and their diagnosis is often challenging.

The present review discusses the clinical and neuroimaging findings of monogenic cSVD, which occurs from the prenatal to adolescent period of development.

## Prenatal period

### *COL4A1/COL4A2*-related disorders

*COL4A1* and *COL4A2*, both located on chromosome 13q34, encode the alpha1 and alpha 2 chains of type IV collagen, a key component of basement membranes in blood vessels and various soft organs in mammals [17]. Mutations in these genes can give rise to brain small vessel disease 1 (BSVD1, OMIM#175,780) and 2 (BSVD2, OMIM#614,483), respectively, and have been associated with a broad spectrum of cerebrovascular, renal, ophthalmological, cardiac, and muscular abnormalities known as *COL4A1/COL4A2*–related disorders. They are inherited in an autosomal dominant (AD) fashion and have a high, de novo mutation rate. This complex of disorders is characterized by highly incomplete penetrance and a wide range of phenotypic variation even among family members [18] and manifest at any developmental stage, from the prenatal (fetal) to the adult stage.

Cerebrovascular manifestations occur in more than half of individuals harboring a *COL4A1* mutation [14]. *COL4A2* mutations are apparently associated with even less penetrance and a globally milder phenotype than *COL4A1* mutations [19]. Seizures are the most prevalent clinical symptom associated with these variants. Motor dysfunction and developmental delay are highly prevalent [18].

**Fig. 1:**
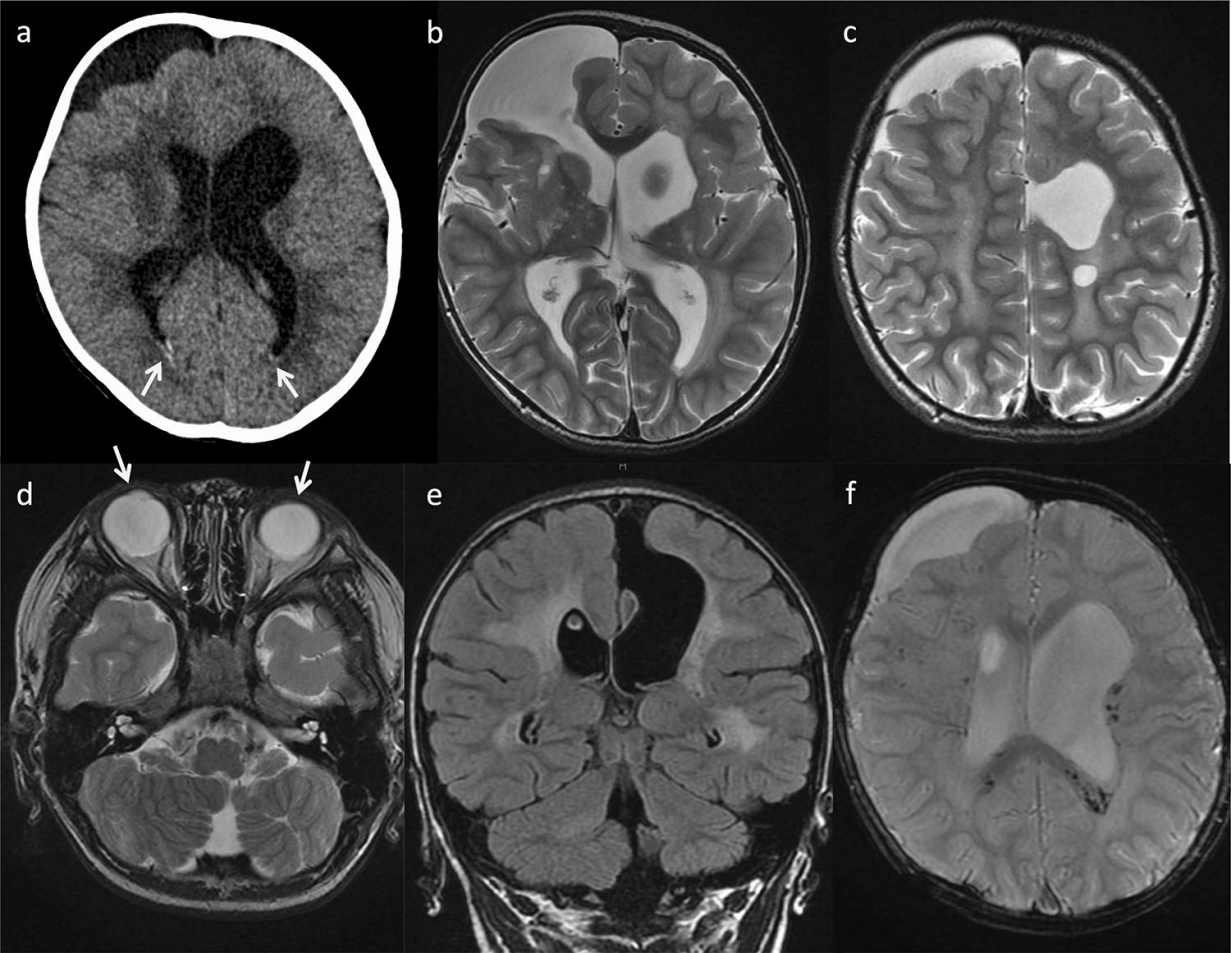
A 3-year-old, male patient with *COL4A1*-related disorder. **a** Axial non-contrast computed tomography demonstrated calcification along the bilateral occipital horns of the lateral ventricles (arrows) and asymmetrical ventricular enlargement. **b-e** Axial T2-weighted imaging and coronal FLAIR imaging revealed porencephaly in the bilateral frontal lobes, asymmetrical ventricular enlargement, hyperintensities in the bilateral basal ganglia, thalami, deep white matter, and cerebellar dysplasia. Note also the implanted intraocular lens for cataracts (arrows). **f** Axial T2*-weighted imaging demonstrated multiple microbleeds in the splenium of the corpus callosum and subcortical and deep white matter

Prenatal and neonatal intracerebral hemorrhage (ICH) and encephaloclastic porencephaly are frequently associated with mutations in both *COL4A1* and *COL4A2* [18, 20–22]. Their most common cause is a germinal matrix hemorrhage leading to deep venous infarction involving the basal ganglia and frontal lobe, tissue necrosis, and porencephalic cavitation (Fig. 1). Prenatal ICH size correlates clinically with motor outcomes [22]. Extensive bilateral porencephaly also resembles hydranencephaly.

Schizencephaly, also known as dysplastic porencephaly, is defined as a cleft extending from the pial surface to the lateral ventricle and lined by heterotopic gray matter. Schizencephaly is also associated with mutations in *COL4A1* and *COL4A2* [23, 24]. Niwa et al. reported susceptibility-weighted imaging (SWI) findings demonstrating hemorrhages in the peripheral portion of the schizencephalic and intraparenchymal region. Venous tortuosity may be helpful in assessing for a possible relationship between schizencephaly and *COL4A1* mutations [25]. Other cortical malformations, ranging from polymicrogyria to subependymal and subcortical heterotopia without hemorrhage or calcification, have also been reported [24].

**Fig. 2:**
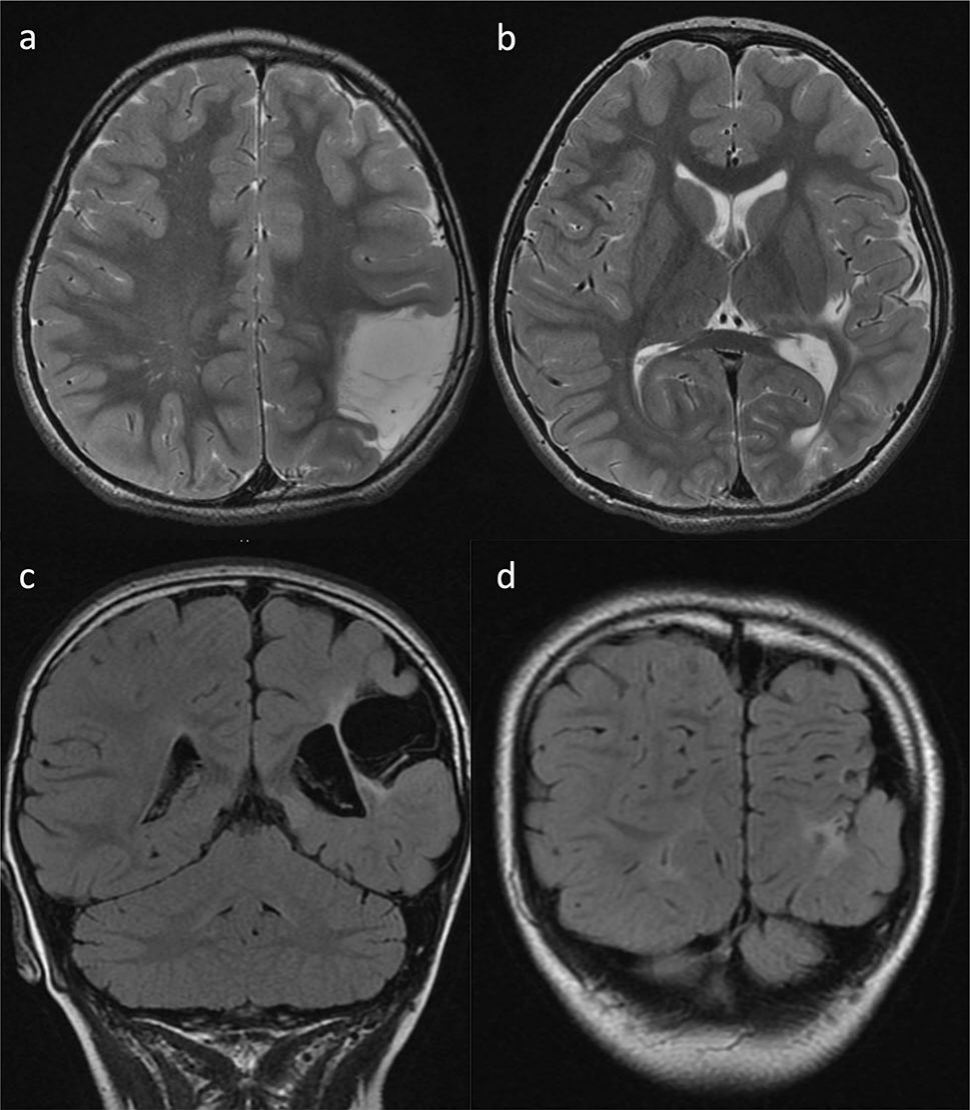
*COL4A2*-related disorder in a 5-year-old, male patient with right hemiparesis. Axial T2-weighted imaging (**a**, **b**) and coronal FLAIR imaging (**c**, **d**) demonstrated cystic encephalomalacia in the left parietal lobe, ulegyria in the left insula and occipital lobe, and hyperintensities in the left posterior limb of the internal capsule and thalamus

Other types of brain lesion related to prenatal hemorrhage have been reported in mutations in children, such as abnormal basal ganglia, dysplastic brain stem, cerebellar hemorrhage, cerebellar hypoplasia/atrophy, brain calcification, ventricular asymmetry, mild ventriculomegaly, and hydrocephalus (Figs. 1, 2) [18, 26, 27] while Dandy-Walker malformation may occur prenatally [28].

Periventricular leukomalacia (PVL), defined as pre- or perinatal, post-hypoxic-ischemic leukoencephalopathy without porencephaly is reported in *COL4A1* mutations [18]. In the absence of a remarkable family history, this PVL phenotype may elude diagnosis. In the absence of other prominent symptoms, such as porencephaly, a high creatine kinase (CK) concentration or microbleeds may be helpful in diagnosing this PVL phenotype in *COL4A1* mutations.

MR angiography can also detect asymptomatic, intracranial aneurysms, dolichoectasia, and vascular tortuosity. These most frequently occur in the internal carotid arteries, specifically in the C4 and C5 segments. In certain instances, they can affect the basilar artery as well [29].

A distinct phenotype known as hereditary angiopathy, nephropathy, aneurysms, and cramps (HANAC) stems from *COL4A1* mutations at exons 24 and 25 [30]. The cerebrovascular phenotype is characterized by cSVD with a low risk of hemorrhagic stroke and the presence of aneurysms around the carotid siphon. Bilateral retinal arteriolar tortuosity is persistent and frequently accompanied by multiple, retinal hemorrhages in the absence of any other ocular abnormality. Patients with HANAC may also experience muscle spasms and kidney lesions, which can contribute to renal cyst formation, chronic kidney failure, and occasionally hematuria.

There is no effective treatment. However, patients and their physicians should be informed that the fragility of the vascular wall may lead to a cerebral or retinal hemorrhage, which can cause the vascular wall to rupture in response to trauma (shock, vigorous physical movement, vaginal delivery) or anticoagulant use, which individuals with this condition should avoid [31]. In pregnant patients with this condition, cesarean delivery has been proposed as a method of avoiding birth trauma to prevent cerebral vascular injury [32].

Similarly, dysfunction of the tight junction components, such as occludin (OCLN) and junctional adhesion molecules (JAMs), is associated with overlapping clinical presentations. In particular, *JAM3* mutations are known to cause congenital cataracts and hemorrhagic destruction of the brain [33, 34]. *JAM3* screening should be requested in prenatal diagnostic screening for congenital cataracts.

### *COLGALT1*-related disorders

*COLGALT1*-related disorders (BSVD3, OMIM #618,360) are autosomal recessive disorders caused by mutations in the *COLGALT1* gene on chromosome 19p13, which encodes the collagen beta galactosyltransferase 1 (ColGalT1) protein. ColGalT1 initiates glycosylation of CoL4a1 (and possibly Col4a2), a crucial step in the formation of the triple helix of collagen IV. Miyatake et al. [35] described two patients with a compound heterozygous variant of the *COLGALT1* gene and demonstrated that decreased ColGalT1 activity leads to decreased synthesis of CoL4a1 protein, thereby reducing type IV collagen secretion. Additionally, Teunissen et al. reported a more severe phenotype with a homozygous essential splice site variant of the *COLGALT1* gene [36].

The resulting phenotype varies highly in terms of the timing and location of intracranial hemorrhages. Some patients may have in utero or early infantile onset accompanied by severe, global, developmental delay, spasticity, and seizures and require full support for daily living. Other patients may exhibit normal or mildly delayed development accompanied by a sudden onset of intracranial hemorrhage resulting in acute, neurological decline. Environmental stress (either in utero stress or an infection) might trigger its onset, as is the case in *COL4A1/COL4A2*-related disease.

**Fig. 3:**
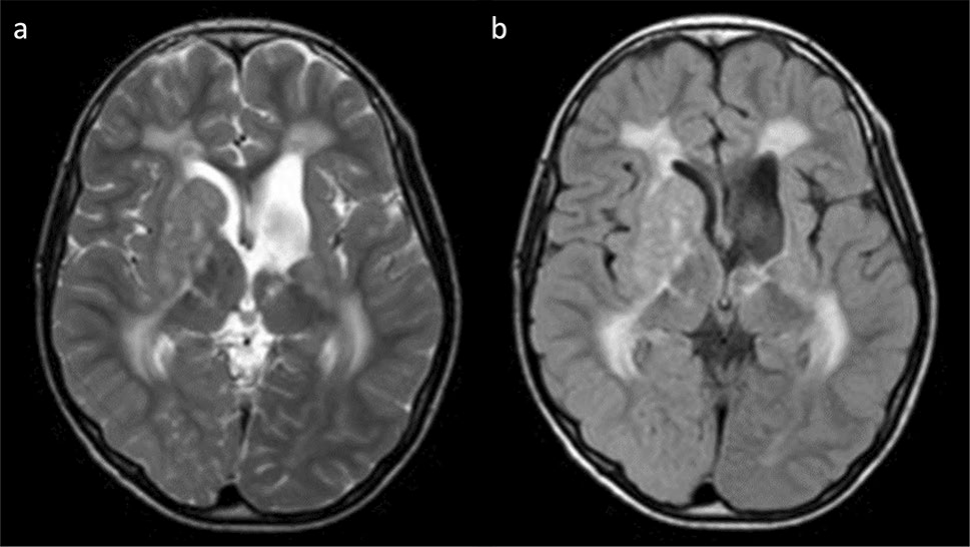
*COLGALT1*-related disorder in a 12-year-old, male patient with severe developmental delay, epilepsy, spastic quadriplegia. Axial T2-weighted imaging (**a**) and FLAIR imaging (**b**) demonstrated porencephaly in the left hemisphere with destructive changes of the basal ganglia and bilateral leukoencephalopathy with mild atrophy. Hyperintense lesions were also observed in the right basal ganglia, posterior limb of the internal capsule, and bilateral thalami (reprinted with permission from Reference [35])

**Fig. 4:**
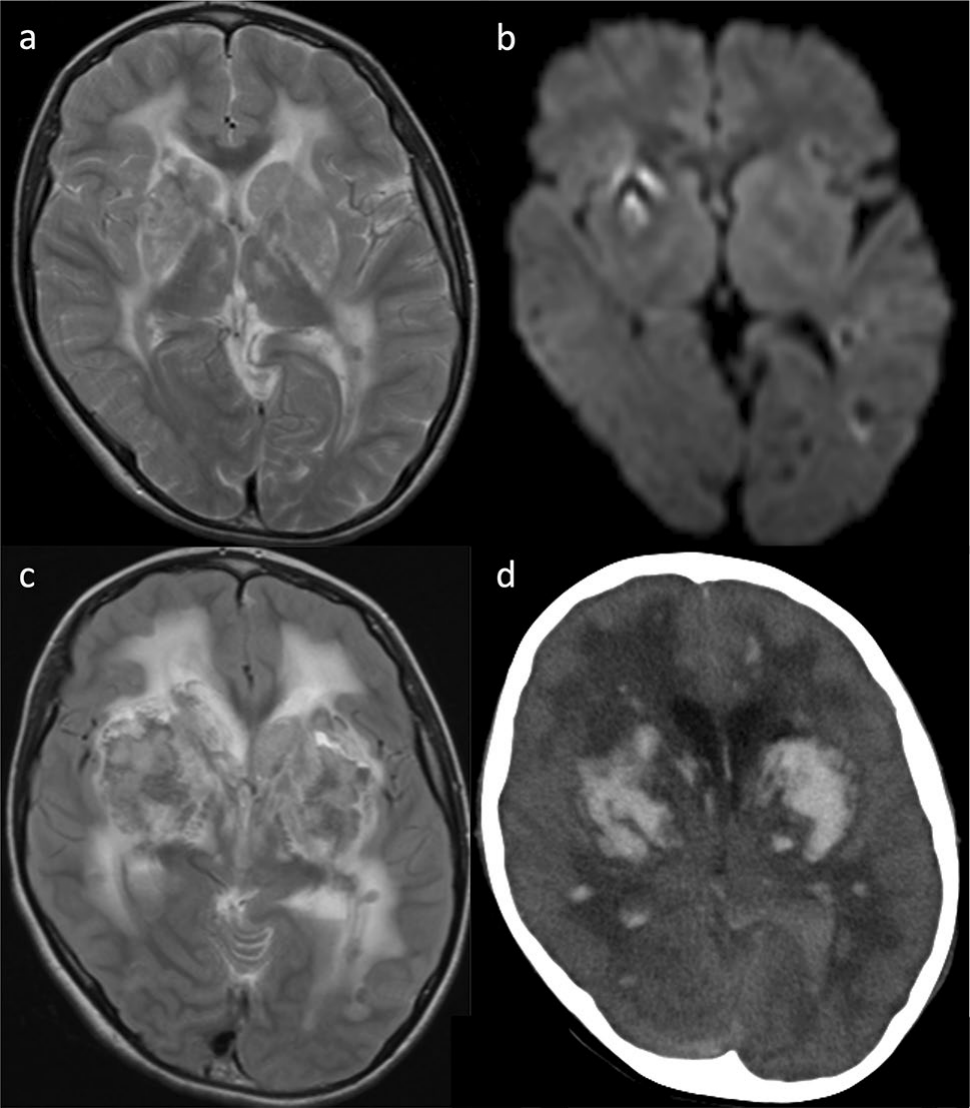
*COLGALT1*-related disorder. Axial T2-weighted imaging (**a**) at the age of 9 years demonstrated hyperintense lesions in the bilateral basal ganglia, thalami, deep cerebral white matter, and internal and external capsules. Axial diffusion-weighted imaging (DWI) (**b**) revealed susceptibility effects of microbleeds in the right basal ganglia and left temporal white matter. After 1 day, axial FLAIR imaging (**c**) and computed tomography (**d**) after the patient lost consciousness owing to an influenza virus infection demonstrated acute, massive, bilateral, parenchymal and intraventricular hemorrhages (reprinted with permission from Reference [35])

The radiographic features of *COLGALT1*-related disorders are also comparable to those of *COL4A1/COL4A2*-related disease [35, 36]. MRI findings typical of *COL4A1/COL4A2*-related disease include porencephaly, parenchymal/intraventricular hemorrhage, hydrocephalus, cerebral calcification, microbleeds, vascular leukoencephalopathy, lacunar infarcts, dilated perivascular spaces, and intracranial aneurysms (Figs. 3, 4).

### Neonatal and infantile stages

#### Incontinentia pigmenti

Incontinentia pigmenti (IP, also called Bloch-Sulzberger syndrome, OMIM #308,300) is an X-linked, neurocutaneous disorder caused by a mutation in the IKK-gamma gene (*IKBKG*), also called *NEMO*, on chromosome Xq28. *IKBKG* is essential for the activation of the nuclear factor-kappa B (NF- κB) transcription factor, which is involved in preventing tumor necrosis factor-alpha (TNF-α)-induced apoptosis and in regulating the immune and inflammatory responses [37]. Thus, *IKBKG*-deficient cells are probably more prone to inflammation and apoptosis. IP is characterized by congenital skin lesions, dental and skeletal dysplasia, and ocular and central nervous system (CNS) abnormalities. IP is almost exclusively seen in females. The mutations appear to be lethal in males; postzygotic mosaicism related to *IKBKG* has been reported in only a few male patients [38].

**Fig. 5:**
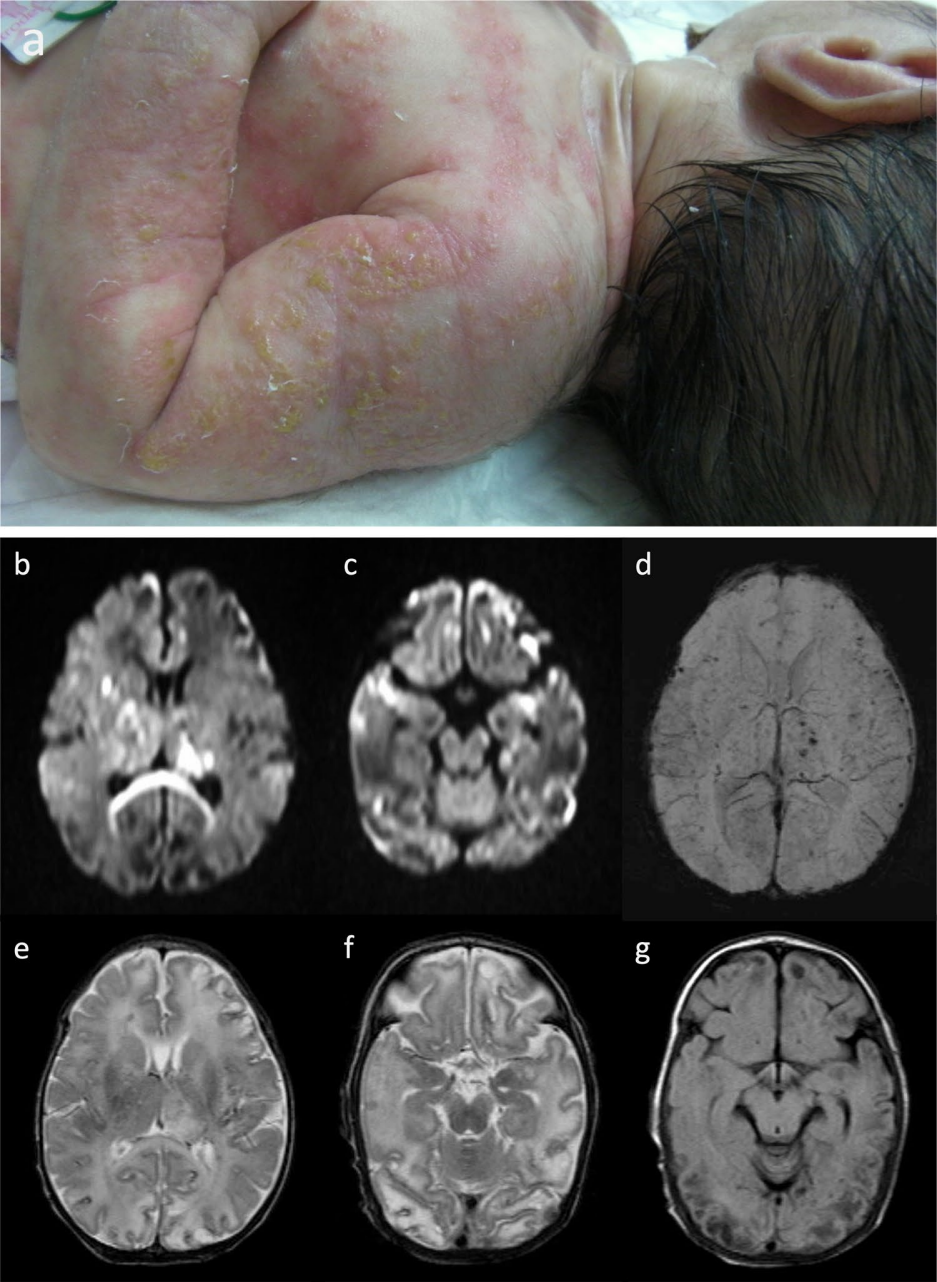
Incontinentia pigmenti in a female neonate with a decreased level of consciousness and weak sucking reflex. **a** A clinical photograph of skin lesions on day 15 shows an erythematous and vesiculobullous rash on her arms and trunk spreading in along the Blaschko lines (reprinted with permission from Reference [40]). **b**, **c** Axial DWI demonstrated multiple areas of ischemic change with scattered foci of restricted diffusion in the bilateral cerebral cortices, basal ganglia, thalami, splenium of the corpus callosum, and midbrain. (**d**) Axial susceptibility-weighted imaging (SWI) demonstrated multiple microhemorrhages in the bilateral cerebral cortices and thalami. **e, f** Axial T2-weighted imaging and FLAIR imaging (**g**) found multiple, cavitary lesions in the bilateral, cerebral subcortical white matter

The skin lesions characteristic of IP progress through the bullous stage (birth to age 4 months); the verrucous stage (for several months); the hyperpigmentation stage (age 6 months to adulthood); to the atretic stage. The skin abnormalities characterizing each stage occur along the lines of embryonic and fetal skin development known as the Blaschko lines [39, 40] (Fig. 5), which correspond to cell migration or growth pathways established during embryogenesis. Similar to dermatomes, they are linear at the extremities and circumferential at the trunk.

Neurological manifestations occur in 30% of IP patients, constituting one of the major causes of morbidity and mortality associated with the condition. Symptoms, such as seizures, intellectual disability, developmental delay, spastic paresis, cerebellar ataxia, and microcephaly, may also be observed. Most neurological features occur from the neonatal through the early infantile period and only rarely in late childhood [41, 42]. Inflammatory mechanisms, vascular injury, and possibly disturbed apoptosis during development are apparently at the root of the cerebral manifestations [43].

Brain MRI can visualize tissue damage resulting from disease in small vessels as well as also medium-sized cerebral arteries [44]. Such tissue damage may include periventricular and subcortical white matter disease, hemorrhagic changes, corpus callosum hypoplasia, polymicrogyria, cortical dysplasia, cerebral atrophy, cerebellar hypoplasia, myelination delays or ventricular dilatation [43, 45]. DWI abnormalities are characteristic, with multifocal and punctate lesions distributed throughout the white matter in a speckled pattern often associated with changes in the corpus callosum. Reduced diffusion is also observed in the basal ganglia, thalami, cerebellum, and cerebral peduncles [40, 46] (Fig. 5). Sequential scans in affected infants demonstrate progressive cortical and white matter cavitation/atrophy, ventricular enlargement, and thinning of the corpus callosum.

Ocular abnormalities are another major cause of disability in IP patients. Approximately 20–37% of IP patients have an ocular defect, such as strabismus, retinopathy, congenital cataract or microphthalmia [47, 48].

Previous studies have reported retinal lesions and perivascular and intravascular eosinophilic infiltration of the CNS and skin in IP. Maingay-de Groof et al. reported that *NEMO* mutation activates eotaxin, a potent, eosinophil-selective chemokine that is highly expressed by endothelial cells in IP and correlates with perivascular and intravascular eosinophilic infiltration [44].

#### Aicardi-Goutieres syndrome

Aicardi-Goutieres syndrome (AGS) typically manifests as an early-onset, subacute encephalopathy usually resulting in profound intellectual and physical disability. AGS presents at birth or in the first few weeks of life with abnormal neurological findings, hepatosplenomegaly, elevated liver enzymes, and thrombocytopenia. Although AGS is phenotypically similar to congenital toxoplasmosis, other, rubella, cytomegalovirus, herpes simplex (TORCH) infections, serological tests for common prenatal infections return negative. Over time, chilblain skin lesions on the fingers, toes, and ears develop in up to 40% of individuals. Severe, neurological dysfunction becomes clinically apparent in infancy, manifesting as progressive microcephaly, spasticity, dystonic posturing, and profound psychomotor retardation, often leading to death in early childhood. Recently, atypical, sometimes milder, cases of AGS have come to light [49].

Many, previous studies have advocated the use of the more generic term, type I interferonopathy (IFN), to refer to this group of monogenic diseases because the upregulation of type I interferon is a crucial aspect of its pathogenesis [50]. Mutations in any of the following nine genes may result in the AGS phenotype: *TREX1* (AGS1, OMIM #225,750), *RNASEH2B* (AGS2, OMIM #610,181), *RNASEH2C* (AGS3, OMIM #610,329), *RNASEH2A* (AGS4, OMIM #606,034), *SAMHD1* (AGS5, OMIM #612,952), *ADAR* (AGS6, OMIM #615,010), *IFIH1* (AGS7, OMIM #615,846), *LSM11* (AGS8, OMIM #619,486), and *RNU7-1* (AGS9, OMIM #619,487). The encoded proteins are involved in nucleic acid metabolism and/or signaling. Spinal fluid and serum analysis reveals elevated levels of interferon activity stemming from increased expression of interferon-stimulated genes in the peripheral blood [51]. *TREX1* mutations are associated with a true neonatal presentation [52]. Most patients present symptoms at a slighter later age. Mutations most frequently occur in *RNASEH2B* [53].

**Fig. 6:**
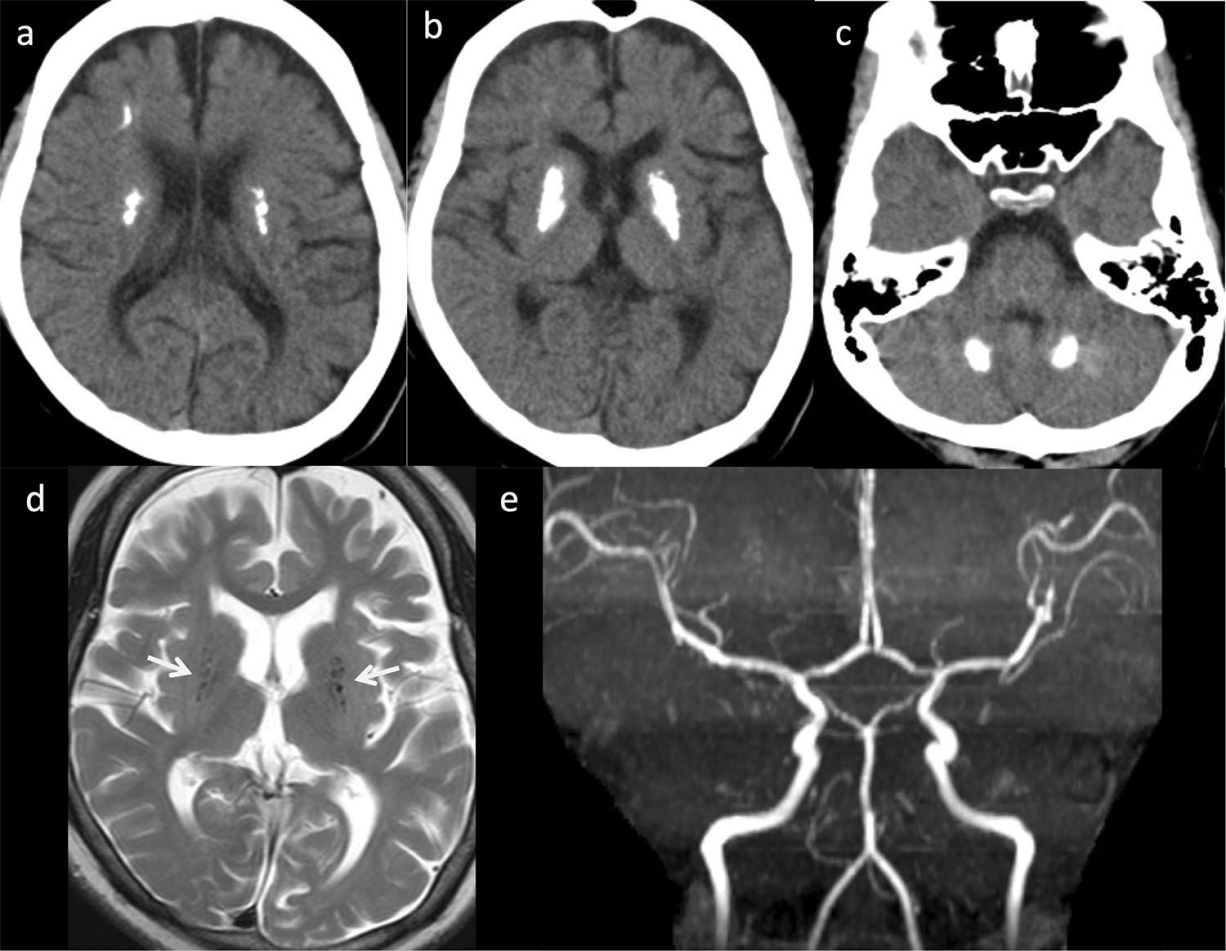
Aicardi-Goutieres syndrome with *IFIH1* mutations in a 15-year-old, female patient with quadriplegia and chilblain skin lesion of the big toe. **a–c** Axial non-contrast computed tomography found multiple calcifications of the bilateral, right frontal, subcortical white matter, bilateral deep white matter, basal ganglia, and cerebellar dentate nuclei. **d** Axial T2-weighted imaging demonstrated mild cerebral atrophy. The bilateral basal ganglia demonstrated a few, punctate hypointensities secondary to calcification (arrows). **e** MR angiography revealed mild stenosis of the major cerebral arteries

Computed tomography (CT) demonstrates multiple, punctate or globular calcifications of the basal ganglia, particularly the putamina, thalami, deep and subcortical white matter, and cerebellar dentate nuclei (Fig. 6). *TREX1* mutations tend to produce particularly severe calcification [53]. T2-weighted imaging demonstrates hyperintensity in the subcortical and deep white matter, especially in the frontal and temporal lobes [49]. White matter rarefaction and anterior temporal lobe cysts are strongly associated with *TREX1* mutations and early age at onset [53, 54].

Cerebral atrophy is usually progressive, and cerebellar atrophy and brain stem atrophy are prevalent. Delayed myelination is associated with *RNASEH2B* mutations and early age at onset [53]. Intracerebral vasculopathy, including intracranial stenosis, moyamoya syndrome, and aneurysms, is associated with *SAMHD1* mutations [55] while bilateral striatal necrosis is associated with *ADAR* mutations [56]. RNASET2-deficient leukoencephalopathy mimics AGS and congenital cytomegalovirus infection clinically and radiologically [57].

Despite the lack of an established treatment, immune modulation (such as corticosteroid therapy) during the active phase may be beneficial [49]. Janus kinase (JAK) inhibitors are reportedly beneficial in controlling inflammation and preventing the progression of end-organ damage by blocking interferon activation in type 1 interferonopathies, including AGS [58, 59].

### Childhood and adolescence

#### Mitochondrial encephalomyopathy with lactic acidosis and stroke-like episodes (MELAS)

Mitochondrial encephalomyopathy with lactic acidosis and stroke-like episodes (MELAS, OMIM #540,000) refers to a heterogeneous group of disorders caused by point mutations in mitochondrial DNA. The majority of patients have in common the pathogenic variant m.3243A > G in the mitochondrial DNA tRNA-leucine (MT-TL1) [60] and may experience the onset of symptoms, including headache, nausea, seizures, episodic vomiting, and permanent or reversible stroke-like episodes as well as some symptoms of generalized mitochondrial disease, at any age (the second decade is the most common). Compared to strokes of vascular origin, there is a higher incidence of clinical symptoms, such as cortical blindness and auditory agnosia [31]. Serum and CSF lactate are usually elevated at the time of presentation.

Although the underlying, pathophysiological mechanism of the stroke-like episodes remains unclear, the cytopathic and angiopathic theories are two, prevailing hypotheses of their etiology [61]. The cytopathic theory proposes that defects in oxidative phosphorylation resulting from mitochondrial mutation cause neuronal and glial cellular dysfunction, potentially resulting in cell death during periods of increased metabolic activity. The angiopathic theory proposes that abnormal mitochondrial function in the arteriolar endothelium leads to impaired autoregulation and ischemia.

**Fig. 7:**
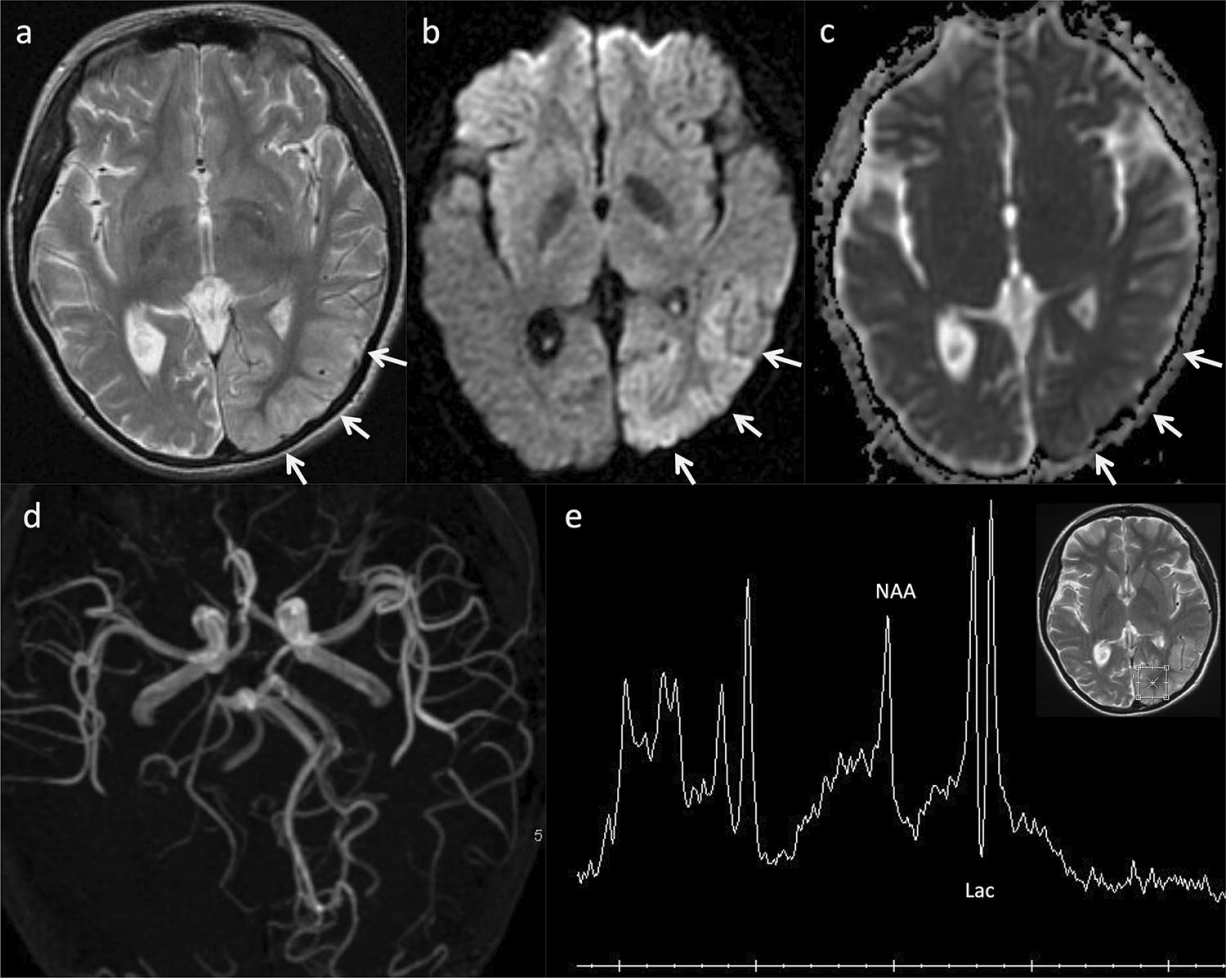
MELAS with point mutations of m.3243A > G in the mitochondrial DNA tRNA-leucine (MT-TL1) in a 10-year-old, female patient with transient visual impairment. Axial T2-weighted imaging (**a**) demonstrated mild swelling and hyperintensity in the left temporal and posterior cortices (arrows). Axial DWI (**b**) demonstrated hyperintensity in the left temporal and posterior regions, and an ADC map (**c**) demonstrated corresponding, increased diffusion (arrows). MR angiography (**d**) revealed dilation of the left middle and posterior cerebral arteries. ^1^H-MRS (PRESS, TE/TR 35/2,000 ms) (**e**) of the left occipital lobe revealed a prominent doublet peak of lactate and decreased NAA/Cr ratio. *MELAS* mitochondrial encephalomyopathy with lactic acidosis and stroke-like episodes, *PRESS* point-resolved spectroscopy, *NAA* N-acetyl aspartate

Brain CT demonstrates symmetrical calcification in the basal ganglia more prominently in older patients [62]. MRI findings of stroke-like lesions do not correspond to vascular territories, involve the cortex and juxtacortical white matter, and primarily affect the parietal and occipital lobes and basal ganglia (Fig. 7). Some studies have reported decreased diffusion in some affected areas [63, 64] while other studies have reported the opposite [65]. Sequential scans may reveal the resolution and subsequent reappearance of abnormal areas or the development of new lesions. Most of the severe lesions progress to cortical laminar necrosis, gliosis, and atrophy [66].

^1^H-MR spectroscopy (MRS) demonstrates a high lactate level in the affected areas of the brain (Fig. 7). The presence of lactate in areas of the brain that are not visibly abnormal on T2 or diffusion imaging is more suggestive of mitochondrial disease [67, 68]. MR angiography reveals prominent dilatation of the arteries (Fig. 7), and perfusion-weighted imaging (PWI) and arterial spin labeling (ASL) demonstrate hyperperfusion in the affected areas in the acute stage, which may be useful in differentiating MELAS from acute ischemic stroke [69, 70]. In the chronic phase, PWI and ASL show hypo- or iso-perfusion in the affected areas [68]. Moreover, regional hyperperfusion has also been revealed on ASL during the preclinical phase three to 5 months before the clinical onset of stroke-like episodes [71].

#### Fabry disease

Fabry disease (FD, OMIM #301,500) is the most prevalent lysosomal storage disorder and is caused by mutations in the *GLA* gene on chromosome Xq22 encoding alpha-galactosidase A (α-GalA). This enzymatic defect leads to abnormal accumulation of globotriaosylceramide (Gb3) in various organ systems, especially vascular endothelial and smooth muscle cells [72]. Renal failure, cardiomyopathy, and CNS alterations are the main causes of morbidity and reduced life expectancy in patients with this disease. Although FD was previously considered to be an X-linked recessive disorder, female patients with heterozygous genetic involvement are known to have a spectrum of presentations ranging from the asymptomatic to the severe. Neonatal screening has detected an unexpectedly high prevalence of this disease, which is diagnosed in approximately 1/3,100 [73], 1/1,250 [74], and 1/7,000 [75, 76] neonates in Italy, Taiwan, and Japan, respectively.

The early pediatric, clinical features of FD are neuropathic pain, anhidrosis, angiokeratoma, and/or cornea verticillate [77]. Neurological involvement, common in adult patients with FD, leads to a wide variety of signs and symptoms, including headache, vertigo/dizziness, transient ischemic attacks, and ischemic strokes. A large, retrospective study of FD patients (*n* = 2446) found that 6.9% of male and 4.3% of female patients with a mean age of 39.0 and 45.7 years, respectively, experienced a stroke [78]. Moreover, patients with stroke had a high prevalence of hypertension, cardiac disease, and renal disease [78].

In FD, both large and small vessels are affected by cerebral vasculopathy. Researchers hypothesize that endothelial dysfunction, cerebral hyperperfusion, a prothrombotic state, and increased synthesis of reactive oxygen species contribute to the vascular dysfunction [79] Additionally, smooth muscle proliferation in the arterial media layer, atherosclerosis, and the initiation of an inflammatory cascade in the vessel wall can worsen vasculopathy in FD [80].

**Fig. 8:**
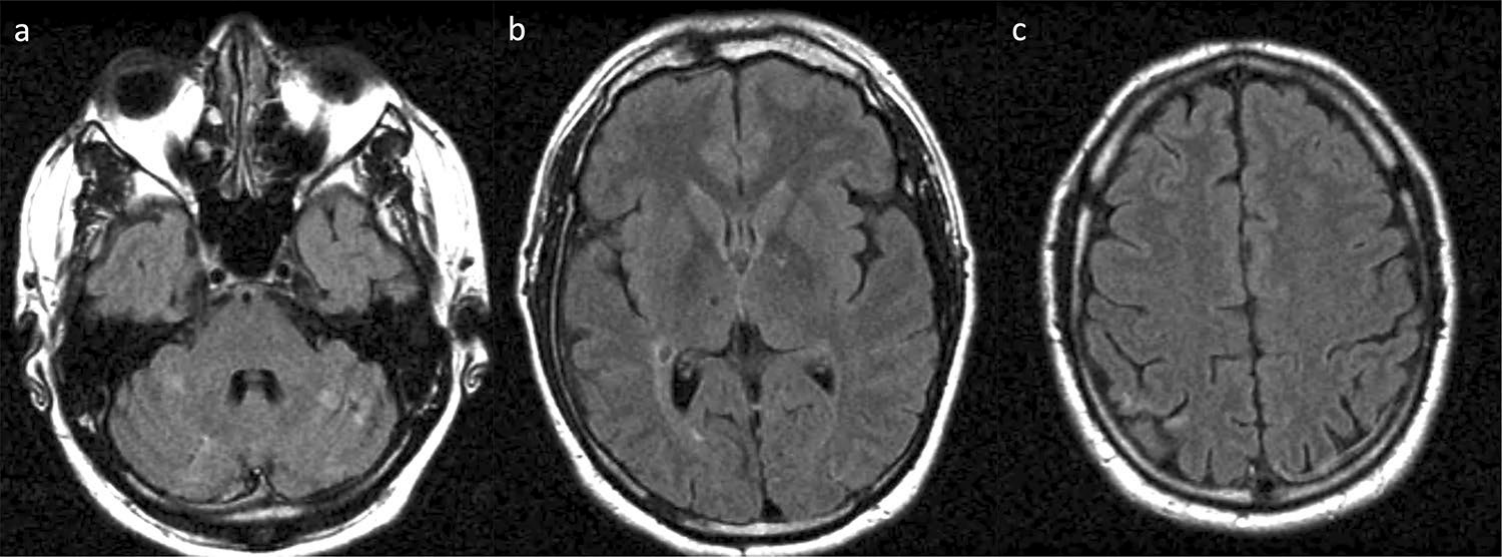
Fabry disease in a 35-year-old, male patient with a history of headache. **a–c** Axial FLAIR imaging demonstrated multiple, small, old infarcts in the cerebral cortices, deep gray matter, deep white matter, and cerebellar hemisphere

Neuroimaging of FD reveals a variety of features ranging from small vessel ischemia, ischemic stroke (including those secondary to cardiac involvement), and hypertensive hemorrhage (including those secondary to renal involvement) to vertebrobasilar artery dolichoectasia (Fig. 8) [77, 81–83]. Deep white matter lesions caused by small vessel ischemia are common in FD even in the absence of neurological symptoms. An accumulation of small vessel ischemia contributes to cognitive decline [84]. Although rare, children and adolescents with FD may present with non-hemorrhagic white matter and deep gray matter lesions alone without any clinical history of stroke [85, 86]. Infarctions in the posterior circulation and vertebrobasilar dolichoectasia reportedly occur at a higher frequency in FD [87, 88].

The pulvinar sign is defined as symmetric or asymmetric hyperintensity in the pulvinar nuclei on T1-weighted imaging. Initially thought to be pathognomonic of the disease, its actual incidence was recently calculated at approximately 3% of FD cases, which is significantly lower than previously thought [89]. Its pathogenesis is still unclear, although the hypothesis that it is caused by subtle, dystrophic calcification stemming from chronic hypoperfusion secondary to microvascular alterations is now widely accepted.

#### Other lysosomal storage disorders

Cerebral arteriopathy has recently been recognized as a feature of other lysosomal storage disorders (LSDs), such as mucopolysaccharidosis (MPS) and Pompe disease.

Mucopolysaccharidoses, a group of inherited LSDs stemming from an accumulation of glycosaminoglycans (GAGs), impact multiple systems. MPS I (Hurler syndrome, OMIM #607,014 and Scheie syndrome, OMIM #607,016) and MPS II (Hunter syndrome, OMIM #309,900) with acute ischemic stroke are the two known types although the number of documented cases is small [90–95]. The disease affects individuals from late infancy to young adulthood, and the infarct occurs in the corona radiata, posterior limb of the internal capsule, and middle cerebral arterial (MCA) territory. Multiple conditions associated with GAG accumulation may contribute to increasing the risk of stroke. Valvular thickening and fibrosis may result in cardiac thrombosis [96]. Severe hydrocephalus may compress the cerebral arteries. Progressive, sclerotic vasculopathy in an MPS I canine model was found to share the morphological and molecular features of massive, eccentric, luminal "plaques" of proteoglycans, collagen, myofibroblasts, vascular smooth muscle cells, and CD68 + activated macrophages with atherosclerosis [97]. Additionally, GAGs and GAG-derived oligosaccharides were found to initiate a pro-inflammatory signaling cascade culminating in macrophage activation via toll-like receptor 4 (TLR4) and its downstream mediator, activated nuclear factor-B (NF-B) [98]. However, more studies are needed to explain the etiology of cerebrovascular arteriopathy in MPS.

Pompe disease (glycogen storage disease type II, OMIM #232,300) is an autosomal recessive LSD characterized by abnormal glycogen accumulation within lysosomes. It is a multisystem disorder involving the heart, skeletal muscle, and liver. Cardiomyopathy and hypotonia are the defining characteristics of infantile-onset Pompe disease (IOPD) whereas involvement of skeletal muscle and respiratory insufficiency predominates in late-onset Pompe disease (LOPD) [99]. Glycogen deposition is found in the neurons of the cerebral cortex, brainstem, and anterior horn of the spinal cord, as well as in the glial cells of the cerebral cortex and the Purkinje cells of the cerebellum [100–102]. Additionally, glycogen deposition also occurs in the peripheral nervous system and smooth muscle of the tunica media of the cerebral arteries [103, 104]. ERT has markedly prolonged efficacy in IOPD but cannot cross the blood–brain barrier; it has also led to the discovery of secondary features of the disease, including white matter abnormalities, subtle signal abnormalities in the basal ganglia and brainstem, and ventricular dilatation in the CNS [105–108]. Brain changes may be the direct result of the glycogen deposition and delayed myelination [105, 108]. Microangiopathies (e.g., white matter lesions, microbleeds, ischemic stroke) and macro-angiopathies (e.g. dolichoectasia, aneurysms, and restrictive arteriopathy mostly of the vertebrobasilar arteries) occur relatively frequently in LOPD [109–113]. Bright tongue sign, an abnormal, diffuse T1 hyperintensity of the tongue musculature, is also common in LOPD [114]. While rare, vertebrobasilar dilative cerebral arteriopathy has also been reported in IOPD [106, 115].

### Cystathionine β‑synthase deficiency (CBSD) (“classic” homocystinuria)

Cystathionine β-synthase deficiency (CBSD, OMIM #236,200), or classic homocystinuria, is an autosomal recessive disorder caused by mutations in the *CBS* gene at 21q22. CBSD impairs cystathionine synthesis and increases homocysteine, methionine, and other metabolites. The eye (ectopia lentis and/or severe myopia), skeletal system (excessive height, long limbs, scoliosis, and pectus excavatum), vascular system (thromboembolism), and CNS (developmental delay/intellectual disability, psychiatric problems, seizures, and/or extrapyramidal signs) are affected [116]. Homocysteine increases superoxide and hydrogen peroxide, coagulation factor, and arterial smooth muscle cell proliferation [117]. Hypercoagulability-related, thromboembolic events can induce occlusive peripheral venous and arterial disorders, cerebrovascular events, and myocardial infarctions, increasing morbidity and death. Hypermethioninemia can serve as a clue to diagnosing CBSD in newborn screening.

**Fig. 9:**
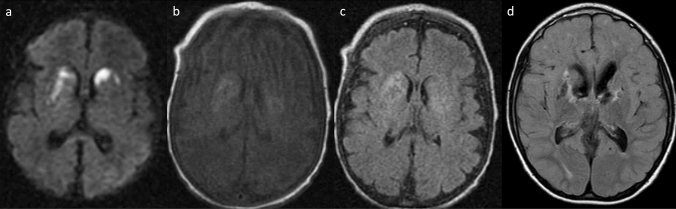
Cystathionine β-synthase deficiency (CBSD) and methylmalonic acidemia in a male neonate with seizure. Axial DWI (**a**) revealed hyperintense lesions in the bilateral caudate nuclei and putamina. Axial T1-weighted imaging (**b**) and FLAIR (**c**) imaging demonstrated mild swelling and hyperintensity in the right caudate nucleus, bilateral putamina, and left globus pallidus indicating necrosis. Follow-up axial FLAIR imaging (**d**) at the age of 9 years found multiple, old infarcts in the bilateral basal ganglia and white matter volume loss

Neuroimaging studies usually reveal several, minor brain infarcts at different ages (Fig. 9) [118]. Secondary venous infarction may result from dural sinus thrombosis [119]. Diffuse signal intensity in the white matter and increased intracranial pressure can result from hypermethioninemia-induced demyelination, white matter vacuolization, and spongy degeneration [120, 121]. MR angiography (MRA) and MR venography (MRV) may help identify large cerebral artery and intracranial dural sinus occlusion, and T2-weighted MRI may demonstrate intraocular lens displacement, another sign of CBSD.

### 5,10‑Methylene‑tetrahydrofolate reductase deficiency (MTHFRD)

5,10-Methylene-tetrahydrofolate reductase deficiency (MTHFRD, OMIM #236,250) is a common inborn error of folate metabolism. It is an autosomal recessive disorder caused by mutations in the *MTHFR* gene, which encodes an enzyme that converts homocysteine to methionine in the remethylation pathway. Thus, MTHFRD causes hyperhomocysteinemia and hypomethioninemia. MTHFRD frequently presents in childhood with gait abnormalities, seizures, and psychomotor impairment [122].

MTHFRD is related to an S-adenosylmethionine (SAM) deficit in the CSF and brain and spinal cord demyelination [123]. *MTHFR* mutations, including a minor form (*C677T* polymorphism), increase the risk of childhood stroke and cervical artery dissection [124]. Patients with MTHFRD should not receive nitrous gas as an anesthetic because it may induce methionine synthase deficiency, which may interact with the hereditary defect in *MTHFR* to cause neurological deterioration and death [125].

**Fig. 10:**
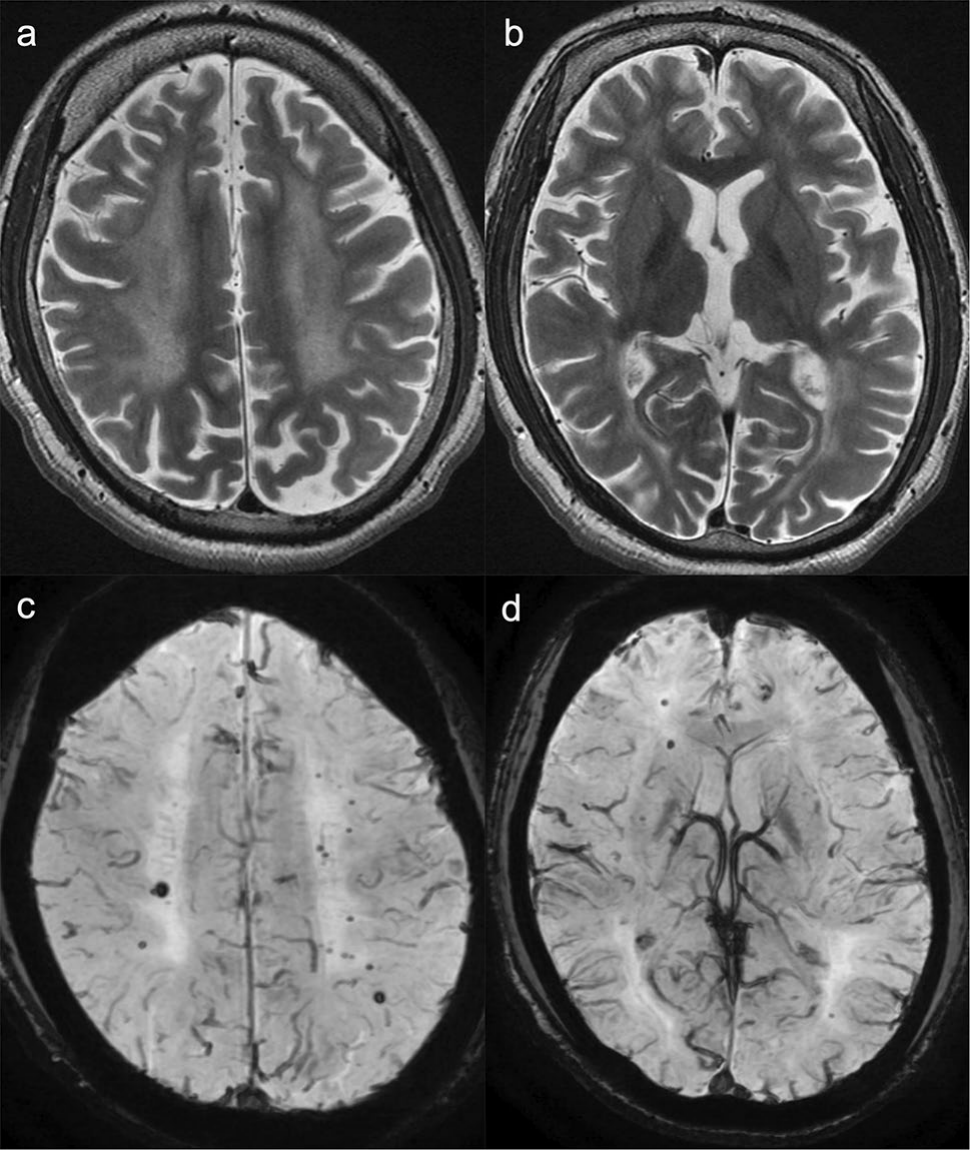
5,10-methylene-tetrahydrofolate reductase deficiency (MTHFRD) in an 18-year-old, male patient with epilepsy, intellectual disability, and an autistic tendency. The patient had lower limb motor weakness and urinary incontinence for 4 years. **a**, **b** Axial T2-weighted imaging demonstrated mild brain atrophy and white matter hyperintensities with posterior predominance. **c**, **d** Axial SWAN demonstrated multiple, cerebral microbleeds in the white matter (reprinted with permission from Reference [121]). SWAN, T2 star-weighted MR angiography

In children and adults with MTHFRD, MRI demonstrates white matter abnormalities ranging from small foci to more widespread regions of high signal intensity indicating demyelination (Fig. 10) [121, 126]. Venous thrombosis, cerebral microbleeds, and vascular infarction may also be present [127]. The dorsal and lateral spinal cord columns may be demyelinated [128]. White matter choline (cho) and N-acetylaspartate (NAA) peak levels on ^1^H-MRS are slightly decreased [129, 130]. Decreased cho is probably secondary to the depletion of labile methyl groups produced by the transmethylation pathway.

### Leukoencephalopathy with calcifications and cysts (LCC)

Leukoencephalopathy with calcifications and cysts (LCC; also known as Labrune syndrome, OMIM #614,561) is a rare, autosomal recessive disorder caused by biallelic mutations in small nucleolar RNA, C/D Box 118 (*SNORD118*), a non-protein-coding, small nucleolar RNA gene on chromosome 17p13 [131, 132]. The clinical presentation of LCC includes slowing of cognitive performance, convulsive seizures (rarely), and a mix of extrapyramidal, cerebellar, and pyramidal signs [133]. Its clinical onset ranges from early infancy to late adulthood. Female patients are slightly more numerous, and the majority of cases are diagnosed in children and young adults [134].

**Fig. 11:**
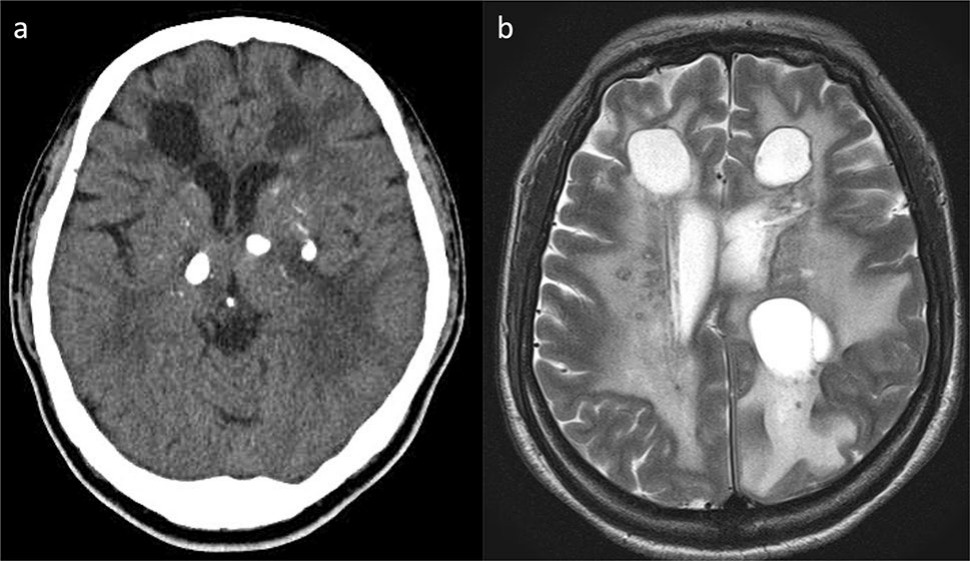
Leukoencephalopathy with calcifications and cysts (LCC) in a 45-year-old, female patient with headache, cognitive decline, spasticity, and speech disturbance. **a** Axial non-contrast computed tomography revealed multiple, asymmetrical calcifications in the bilateral basal ganglia, thalami, and subcortical white matter and cyst formation in the bilateral, frontal white matter. **b** T2-weighted imaging demonstrated multiple, giant cysts and leukoencephalopathy, along with multiple, punctate hypointensities reflecting the calcifications in the bilateral white matter (this case was provided by professor Junichi Takanashi of Tokyo Women’s Medical University, Yachiyo Medical Center)

LCC is characterized by a triad of neuroimaging findings, including diffuse and asymmetric leukoencephalopathy, extensive calcification, and parenchymal brain cysts (Fig. 11). CT and MR imaging demonstrate increased white matter signal intensity sparing the U-fibers and corpus callosum, with extensive, coarse calcification of the basal ganglia, brain stem, and subcortical white matter. Ring-like enhancement along the cyst wall or nodular enhancement in the degenerated white matter indicates disruption of the blood–brain barrier (BBB) and angiogenesis [134, 135]. DWI reveals increased diffusion in the cysts and surrounding white matter, indicating excessive water content. ^1^H-MRS of the cysts demonstrates only the lactate peak but no brain metabolites [135], indicating energy failure in the cyst wall parenchyma. Furthermore, Wang et al. reported reduced NAA and cho in the leukoencephalic lesions [136].

Histopathological examination reveals vascular, tumor-like hyperplasia, Rosenthal fibers, glial proliferation, calcification, hyperplasia of the vessel wall, necrosis, degeneration, iron deposition, demyelination, vascular inflammatory changes, thrombosis, and hemorrhage [133–135].

The neuroimaging features of LCC are similar to those of cerebroretinal microangiopathy with calcifications and cysts (CRMCC, OMIM #612,199), also known as Coats plus syndrome. In contrast to LCC, CRMCC is also characterized by extraneurological, systemic lesions, such as bilateral retinal telangiectasias and exudations, intrauterine growth retardation, skeletal and hematological abnormalities, recurrent gastrointestinal hemorrhage, sparse hair, and dysplastic nails. Although LCC and CRMCC were initially thought to be manifestations of the same disorder, recessively inherited compound heterozygous mutations in the *CTC1* gene have been identified in CRMCC patients, suggesting that these two disorders are not allelic [137, 138].

### Deficiency of adenosine deaminase type 2 (DADA2)

Deficiency of adenosine deaminase type 2 (DADA2, OMIM #615,688) is an autosomal recessive disorder caused by a mutation in the *ADA2* gene (previously known as *CECR1*) on chromosome 22q11. ADA2 is a dimeric, extracellular enzyme primarily secreted by myeloid lineage cells that converts adenosine to inosine. In addition to its catalytic function, ADA2 is thought to possess anti-inflammatory and immunomodulatory properties and to contribute to the maintenance of vascular integrity [139].

DADA2 usually presents in childhood. Most patients exhibit symptoms of a systemic, vascular, inflammatory disorder, including skin ulceration and recurrent strokes, which lead to neurological dysfunction. Additional characteristics may include recurrent fever, elevated acute-phase proteins, myalgia, lesions resembling polyarteritis nodosa, and/or livedo racemosa or reticularis with inflammatory vasculitis on biopsy. Some patients may exhibit renal and/or gastrointestinal involvement, hypertension, aneurysm formation, ischemic necrosis of the digits or clinical immunodeficiency. The hematological manifestations may sometimes resemble Diamond-Blackfan anemia [140].

**Fig. 12:**
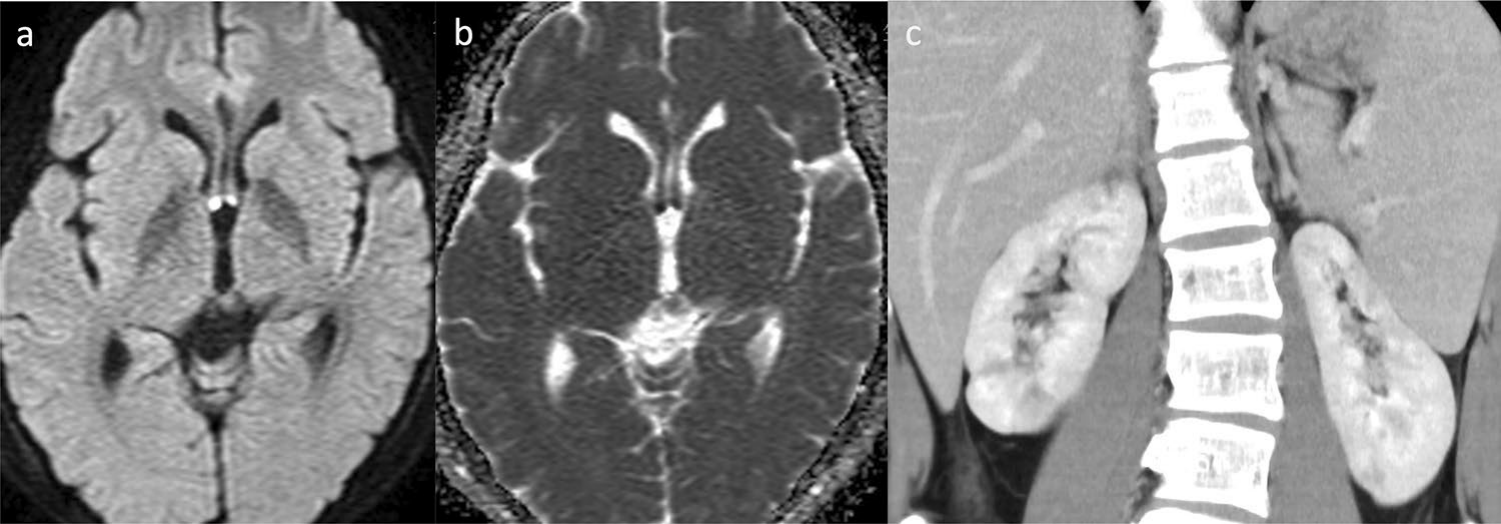
Deficiency of adenosine deaminase type 2 (DADA2) in a male patient aged 18 years who received the diagnosis of DADA2 at the same time as his older brother (this patient has already been reported by Nihira et al. “P3” corresponds to this case). [142] Axial DWI (**a**) and ADC map (**b**) exhibited diffusion restriction in the bilateral column of the fornix, leading to the diagnosis of fornix infarction. The patient experienced memory loss spanning 2–3 days prior to MRI imaging. MR angiography found no stenosis in the major branches (not shown). Contrast-enhanced computed tomography (**c**) demonstrated impaired renal cortical enhancement and irregularities suggestive of a previous renal infarction. Additionally, splenomegaly was observed

**Fig. 13:**
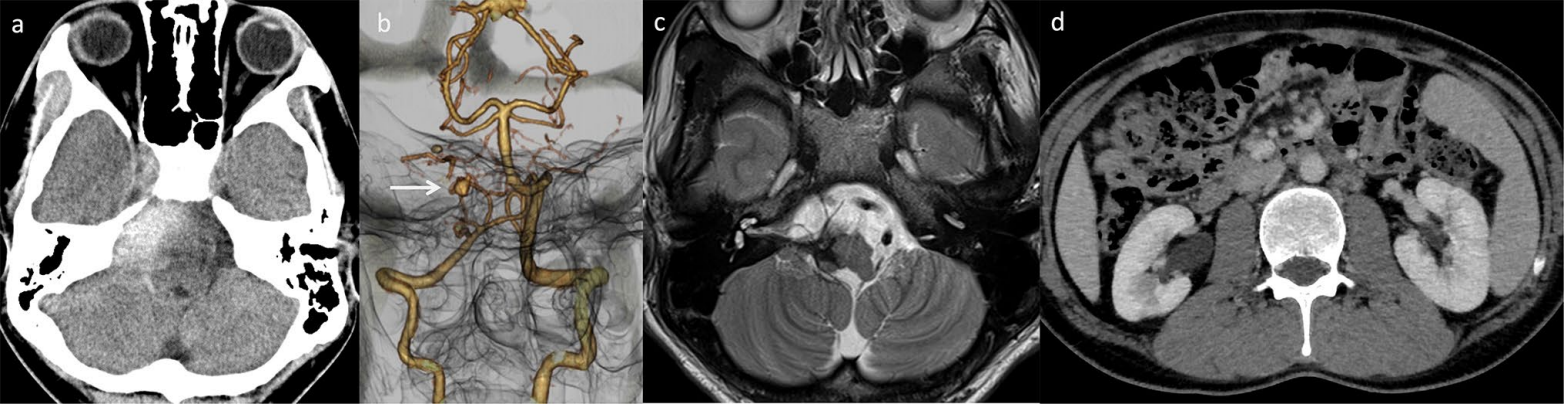
A 26-year-old, male patient, the sibling of the patient mentioned above (Fig. 12), with deficiency of adenosine deaminase type 2 (DADA2). He had a history of recurrent diplopia, sensory numbness, and right-sided hearing impairment since the age of approximately 10 years. His cutaneous symptoms consisted of rash (livedo racemosa) and a history of frostbite. At the age of 22 years, DADA2 caused by defective ADA2 activity in the plasma was diagnosed (this patient has already been reported by Nihira et al. “P2” corresponds to this case) [142]. **a** Initial head non-contrast computed tomography (CT) revealed a parenchymal hemorrhage on the right side of the pons and a subarachnoid hemorrhage along the prepontine cistern. **b** Subsequent CT angiography indicated a possible pseudoaneurysm of the right anterior inferior cerebellar artery (AICA) (arrow). **c** Follow-up T2-weighted imaging demonstrated hemosiderin deposition around the brainstem region, suggesting previous, hemorrhagic events. **d** Contrast-enhanced CT highlighted impaired renal cortical enhancement and irregularities suggestive of a previous renal infarction. Splenomegaly was also observed

CNS involvement resulting from small- and medium-sized vessel vasculopathy is a well-recognized complication occurring in approximately 50% of cases [141]. Neuroimaging features of DAD2 are multiple, recurrent, acute or chronic lacunar ischemic infarcts located in the deep brain nuclei and/or the brainstem (Fig. 12) [142–144], hemorrhagic stroke, intracranial bleeding (Fig. 13) [142–144], and spinal infarcts [143]. MR angiography may reveal an aneurysm or stenosis in the medium-sized arteries [143, 144]. Peculiar, probably inflammatory, perivascular tissue in the basal and prepontine cisterns may also be observed [143]. Peripheral neuropathy, acute sensorineural hearing loss, optic neuritis, and other ophthalmological abnormalities may also occur.

Anti-tumor necrosis factor (TNF) agents are recommended as a treatment for both symptomatic and asymptomatic DAD2 as they prevent or eliminate manifestations of autoinflammatory disease and vasculitis, reduce the risk of ischemic stroke, reduce the inflammatory burden, and ameliorate immunodeficiency, hepatosplenomegaly, and neutropenia [140]. HSCT has been shown to be curative for DADA2 [145].

## Conclusion

The present review discussed the clinical and neuroimaging features of monogenic cSVD, which occurs from the prenatal to adolescent stage of development. A multidisciplinary approach involving pediatric neurologists, geneticists, and neuroradiologists is essential for accurate diagnosis, comprehensive monitoring, and tailored management of monogenic cSVD. Advancing our understanding of these disorders is essential for improving clinical outcomes in affected individuals.
